# Colour Stability, Fatty Acid Profile, and Lipid Oxidation in Meat Stored in Modified Atmosphere Packaging from Light Lambs Fed with Concentrate with Carob Pulp (*Ceratonia siliqua* L.)

**DOI:** 10.3390/antiox12081482

**Published:** 2023-07-25

**Authors:** Diego Nicolas Bottegal, Sandra Lobón, María Ángeles Latorre, Juan Ramón Bertolín, Javier Álvarez-Rodríguez

**Affiliations:** 1 Departament de Ciència Animal, Universitat de Lleida, 25198 Lleida, Spain; 2 Departamento de Ciencia Animal, Centro de Investigación y Tecnología Agroalimentaria de Aragón (CITA), 50059 Zaragoza, Spain; slobon@cita-aragon.es (S.L.); jrbertolin@cita-aragon.es (J.R.B.); 3 Instituto Agroalimentario de Aragón—IA2, CITA-Universidad de Zaragoza, 50059 Zaragoza, Spain; malatorr@unizar.es; 4 Departamento de Producción Animal y Ciencia de los Alimentos, Universidad de Zaragoza, 50013 Zaragoza, Spain

**Keywords:** lamb, carob pulp, fatty acid, malondialdehyde, condensed tannin, α-tocopherol, *Semimembranosus*

## Abstract

There is a growing interest in using by-products rich in polyphenols, such as carob pulp (Cp, *Ceratonia siliqua* L.), as a dietary source of antioxidants for animals. This study assesses the effects of including Cp in lambs’ diet and meat display time (0, 7, 9, and 11 days) in modified atmosphere packaging on meat colour, fatty acid (FA) composition, tocopherol levels, and lipid oxidation values in the *Semimembranosus* muscle of 40 light lambs. The lambs were fed with concentrates supplemented with increasing Cp levels (0, 150, and 300 g/kg) for 45 days before slaughter. Metmyoglobin (MMb) and malondialdehyde (MDA) contents increased linearly with display time (*p* < 0.05), regardless of diet (*p* > 0.05). At 11 days of display, MMb (28 ± 0.8%) and MDA (0.6 ± 0.1 mg MDA/kg of meat) contents remained within the acceptable limits. The α-tocopherol content was lower in the 30% Cp group and meat (*p* < 0.05). Total saturated and monounsaturated FA contents (934 ± 64 and 823 ± 65 mg/100 g of meat, respectively) did not differ significantly among the groups. However, the meat from lambs fed with 30% Cp showed reduced levels of branched-chain FAs, while polyunsaturated FAs increased (*p* < 0.05) compared to the control lambs. The inclusion of Cp in the lamb’s diet, up to 30%, did not lead to meat deterioration and improved certain quality parameters, including a healthier FA profile. These findings highlight Cp’s potential as an alternative antioxidant source in animal diets.

## 1. Introduction

During the storage of retail meat cuts, the main problem is the biochemical deterioration of the meat, which is especially caused by oxidative reactions and microbial proliferation. These processes are reflected in meat discolouration, unpleasant flavours and odours, and toxic compound production [1], representing both a potential public health hazard and economic losses. At present, modified atmosphere packaging (MAP) is a useful tool to provide a stable bloomed ‘bright-red’ colour in lamb meat and prolong its shelf-life [2]. However, over time, its high O_2_ concentration may lead to an increase in lipid oxidation values. If meat is enriched with polyunsaturated fatty acids (PUFAs), it is more predisposed to oxidation and the production of oxygen free radicals, aldehydes, and ketones [3]. This process can also accelerate the oxidation of proteins, such as myoglobin, which results in meat browning [2].

There is an increasing interest in the inclusion of carob pulp (Cp, *Ceratonia siliqua L*.) in animal diets due, in part, to the high level of secondary compounds (phenolic acids, flavonoids, saponins, and condensed tannins (CTs)). CTs have functional activities and provide health benefits (such as antioxidant, anti-inflammatory, and anti-aging properties) [4]. Although previous studies highlighted the effects of CTs on lambs’ performance [5], more attention has recently been paid to its effects on meat quality [6]. The literature is not completely conclusive about the effects of tannins on meat; however, some studies highlight an improvement in meat-colour stability [7], lower lipid oxidation [8], a higher fatty acid (FA) concentration with human health benefits [9,10], and a reduction in skatole subcutaneous caudal fat and typical sheep meat odour [11]. At present, the consumer rejection of non-natural antioxidants in the meat industry has led to the examination of other antioxidant sources [12]. For example, vitamin E is a natural antioxidant highly used in lamb diets to increase the meat shelf-life, but Cp may be a source of CTs, which can replace outsourced and/or synthetic antioxidants [13,14].

On the other hand, increasing the concentrations of some fatty acids, such as conjugated linoleic acid (CLA) isomers and vaccenic acid (VA, *trans*11-C18:1), in products derived from ruminants has also been of interest to the meat industry, due to its beneficial effects on health [15]. In line with this, it was demonstrated that dietary polyphenols (including CTs) may interfere with the ruminal biohydrogenation (BH) process and, consequently, modulate the FA profile of ruminant products [6,16]. However, to our knowledge, the effects of Cp on light lamb meat FA composition are still unclear, as they also depend on the remaining dietary components (forage or concentrate-based) and their nutrient composition. 

This study aims to evaluate the effect of the inclusion of Cp (0, 150, and 300 g/kg of concentrate) in the diet of lambs and meat display time (0, 7, 9, and 11 days) in MAP on the meat-colour attributes, antioxidant content, FA composition, and lipid oxidation.

## 2. Materials and Methods

All procedures employed with animals in this study followed the Spanish Animal Protection Regulations RD 53/2013, which complies with the European Union Directive 2010/63 regarding the protection of experimental animals. The animal procedure protocol was supervised by an in-house Animal Experimentation Committee (code CEEA 01-03/21).

### 2.1. Diets, Slaughter Procedures, and Meat Sampling 

In the present experiment, 40 carcasses from crossbred (Romane × Berberine × Ripollesa) uncastrated male lambs with an average body weight (BW) of 27.3 ± 3.8 kg and age of 83.2 ± 9.3 days were used, which were selected from two consecutive fattening batches (winter and summer) of 72 animals each. The lambs’ fattening period lasted 45 days per batch, during which the animals were randomly allocated into group pens (6 animals/pen) and received one of the three fattening experimental diets (4 pens/dietary treatment/batch). The lambs were fattened during January–February and June–July, in the winter and the summer batch, respectively. Experimental diets were defined according to the Cp inclusion level and were formulated to be isonitrogenous (17.5% crude protein) and isoenergetic (7.36 MJ of net energy for ruminants/kg of concentrate) diets. The Cp replaced grains and co-products in the concentrate mixture at inclusion levels of 0, 150, and 300 g/kg of the concentrate, for treatments C0%, C15%, and C30%, respectively. Water, concentrates (including the control diet, 28% maize; 28.9% barley; 8% wheat; 4% wheat bran; 12% gluten maize feed; 14.4% soybean meal; 0.9% palm oil; 2.5% calcium carbonate; 1% salt; and 0.3% vitamin–mineral premix), and barley straw were always available ad libitum. The FA composition and tocopherol and polyphenol contents were analysed and are described in Table 1. A more detailed explanation of the experiment design, farm animal management, and nutritional composition of the diet was previously described by Pelegrin-Valls et al. [17].

At the end of the fattening period and after 3–4 h of a fasting period, all lambs were transported 3 km from farm to the BonÀrea abattoir the same day within each batch. After slaughter, a total of 19 carcasses from the winter batch (5–7 carcass/diet) and 21 from the summer (7 carcass/diet) were selected according to the average BW. Thus, a total of 12, 14, and 14 carcasses for the C0%, C15%, and C30% treatment were selected, respectively.

**Figure 1:**
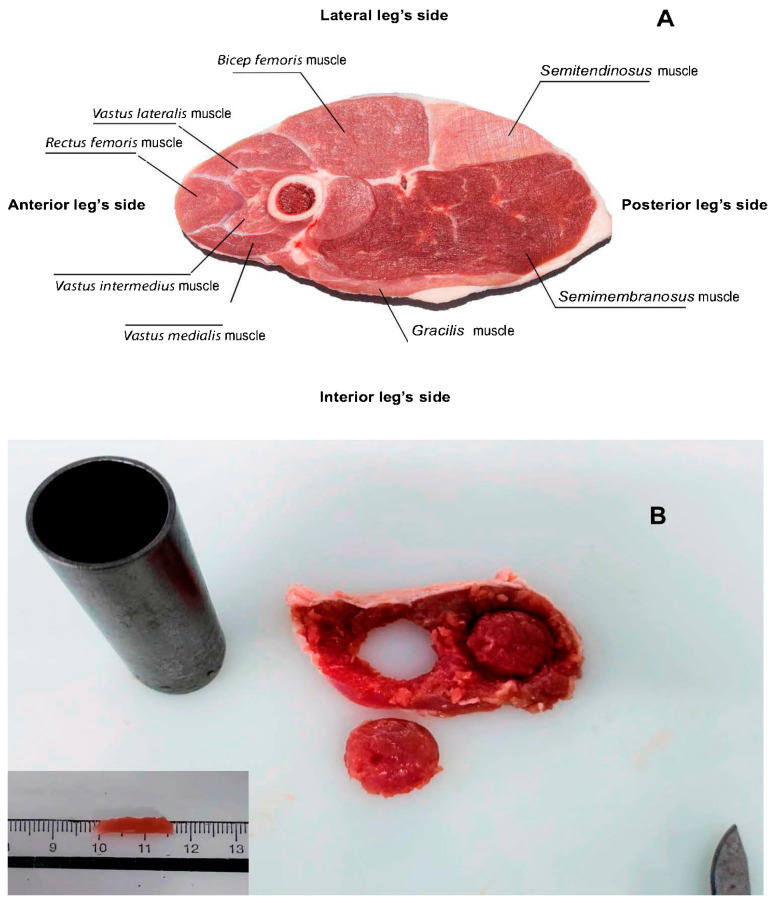
Light lamb chop muscle composition (**A**) and sampling of *Biceps femoris* muscle for drip loss measurement (**B**). The two pieces had a 2.5 cm diameter and one had 15 mm × 3 mm × 3 mm (in the lower left corner).

After a chilling period of 18 h at 4 °C, the carcasses were jointed and four 1.5 cm thick chops (100 g approx. each, Figure 1A) were obtained from the right leg to be randomly assigned to four display times (0, 7, 9, or 11 days). All samples, excluding the 0 d chops, were packed under a high-oxygen MAP (80% O_2_ + 20% CO_2_, TSA 680 traysealer, ULMA, Guipúzcoa, Spain). Transparent polyethylene trays were used and wrapped with oxygen-permeable polyamide/ethylene-vinyl alcohol copolymer/polyethylene (PA/EVOH/PE) laminate film (thickness 45 µm, water vapour transmission rate at 38 °C of 18 g/m^2^/24 h/98% relative humidity (RH), O_2_ transmission rate of 5 cm^3^/m^2^/24 h and CO_2_ transmission rate of 25 cm^3^/m^2^/24 h at 23 °C, 50% RH and 1 atm, OPALEN HB 45 AF, Amcor flexibles, Granollers, Spain). All trays were kept in the dark at 4 ± 1 °C during the display time stipulated and every tray contained three chops (one of each dietary treatment), which were placed above absorbent pads.

### 2.2. Thawing and Drip Loss, Moisture Content, and Colour Stability Measurements

At 18 h post-slaughter for the 0 d samples and at the end of the display storage period for the 7, 9, and 11 d samples, the meat colour was evaluated by measuring the colour parameters L*, a*, and b* after 1 h of blooming. Colour attributes were measured in the CIELab space on the *Semimembranosus* muscle (SM) from each chop in duplicate, using a portable Minolta CM-700d spectrophotometer (Konica Minolta Sensing Inc., Osaka, Japan) with a measurement area diameter of 8 mm, including a specular component and a 0% UV, standard illuminant D65 (colour temperature of 6504 K), observer angle 10° and 0. The reflectance spectra from 400 to 700 nm wavelength were recorded for the calculation of metmyoglobin (MMb) formation, following the equation:$\;\mathrm{MMb}\;\left(\%\right)=100\times \left\{1.395-\left[\left(A572\mathrm{nm}-A700\mathrm{nm}\right)/\left(A525\mathrm{nm}-A700\mathrm{nm}\right)\right]\right\}$ [18]. In addition, colour saturation (C*) and hue angle (h°) were calculated as: $C^{*}=\left(\sqrt{a^{*2}+b{*}^{2}}\right)$ and h°=57.29×tan−1b*a*, expressed in degrees. Following the colour assessment, the samples were weighed, vacuum-packed, and frozen (−80 °C) for subsequent analysis. 

Chops were allowed to thaw for 24 h (at 4 °C) and were subsequently deboned and trimmed of subcutaneous fat and epimysium; thus, the SM (Figure 1A) was divided into two pieces. One was used for the lipid oxidation analysis and the other for FA and tocopherol content analyses, which was freeze-dried (Freeze-dryer gamma 2–16 LSCplus, Martin Christ, Osterode am Harz, Germany). The thawed losses were calculated as: 100 × (pre-frozen chop weight − thawed chop weight)/pre-frozen chop weight. Meat moisture was determined by the difference between the post-frozen and freeze-dried weight.

All measurements were conducted in two different muscles to overcome the analytical weight requirement constraints. Thereby, drip loss was estimated by two methods in the *Biceps femoris* muscle of each chop, so 2 pieces of 1 cm thick × 2.5 cm in diameter were taken (Figure 1B) to perform the EZ drip loss method [19]. However, no appreciable amount of juice was collected in the EZ container, and thus no reliable data were obtained. Additionally, three 15 mm × 3 mm × 3 mm (0.2 g) replicates were obtained and submitted to a centrifugation process to estimate the drip loss, according to the method described by Kristensen et al. [20].

### 2.3. Analysis of Fatty Acid Composition in Feedstuffs and Semimembranosus Muscle Sample

The FA profile of feedstuffs and intramuscular fat (IMF) of SM at day 0 of display were determined using gas chromatography with a flame ionization detector (FID). The FA composition of feedstuffs was extracted according to Sukhija and Palmquist [21] using C19:0 as an internal standard and the FA of meat samples were extracted according to Lee et al. [22] using C23:0 as an internal standard. Briefly, 500 mg of both lyophilized samples were minced and mixed with 3 mL of heptane and 4 mL of NaOH/CH_3_OH 0.5 M. The mixture was homogenized with a vortex and heated with agitation for 20 min at 70 °C for feed samples and for 20 min at 50 °C for meat samples, followed by 6 min of cooling. Then, 4 mL of acetyl chloride/CH_3_OH (1/10 *v*/*v*) was added. The mixtures were vortexed and reheated with agitation (orbital shaker) for 100 min at 70 °C and 60 min at 50 °C, respectively. After cooling to ambient temperature (25 °C), 2 mL of ultrapure water was added. Then, the mixture was shaken (orbital shaker) and homogenized for 10 min, and centrifuged for 5 min, 3500 rpm at 10 °C. Later, the upper layer (heptane) was taken and transferred to 5 mL tubes with anhydrous Na_2_SO_4_ to remove traces of water. In the case of feeds, activated carbon was added (to remove interfering compounds, such as pigments). Additionally, this mixture was shaken for 10 min. Later, both samples were centrifuged for 5 min, 3500 rpm at 10 °C. Finally, 1 mL of the supernatant was carefully transferred into an amber screw-cap glass vial for gas chromatography. The FA methyl esters (FAMEs) were analysed using a Bruker SCION 436-GC (Bruker, Billerica, MA, USA), equipped with a CP-8400 autosampler, an SP-2560 capillary column (200 m × 0.25 mm × 0.2 µm film thickness), an FID detector, and a CompassCDS software (v. 3.0.1). The FAs were identified using several commercial FAME standards (GLC-532, GLC-401, GLC-642, GLC-643, GLC-538, and GLC-463, Nu-Chek, Elysian, Chicago, IL, USA) and as recommended in the literature [23,24,25]. The FAMEs were quantified following the indications of ISO [26]. The ether extract in the feed was determined with an XT10 Ankom extractor (Ankom Technology Corporation, Fairport, NY, USA). The IMF content was calculated as the sum of each individual FA detected, expressed as the triglyceride equivalent [27].

### 2.4. Analysis of Cholesterol, Tocopherol Isomers, and Total Polyphenols

On feedstuff samples, tocopherol isomers (α, γ, and δ-tocopherols) were analysed by liquid chromatography according to the methodology described by Blanco et al. [28] Additionally, cholesterol and the same tocopherol isomers were analysed in the meat at day 0 of display, following the methodology described by Bertolín et al. [29]. Briefly, 200 mg of the freeze-dried feed samples were extracted three times with 3 mL of methanol–acetone–petroleum ether (1:1:1 *v*:*v*:*v*, 0.01% *w*/*v* of 2,6-di-tert-butyl-4-methylphenol (BHT) in ethanol). Then, all the extracts were evaporated in a vacuum evaporator, and the dry residue was obtained. For the meat samples, 200 mg of freeze-dried meat were subjected to an overnight saponification process with 200 mg of L-ascorbic acid and 3 mL of saponification solution (10% *w*/*v* potassium hydroxide in ethanol:distilled water in a 50:50 *v*:*v* mixture) in an orbital shaker (600 rpm) at 25 °C. Then, the extraction was conducted with 5 mL of *n*-hexane:ethyl acetate 9:1 *v*:*v* and 5 µg mL^−1^ of the BHT mixture. The mixture was vortexed, shaken in an orbital shaker (600 rpm) for 15 min, and subsequently centrifuged at 2000× *g* at 10 °C for 5 min. The recovered upper layer (organic solution) was evaporated in a rotational vacuum concentrator at 40 °C for 30 min. Finally, both evaporated residues (feed and meat) were dissolved in 1 mL of mobile phase acetonitrile:methanol:dichloromethane (75:15:10 *v*:*v*:*v*), shaken in an orbital shaker (600 rpm) for 10 min at room temperature, and filtered into a 2 mL amber screw-cap vial for liquid chromatography. The extracts were injected into an ACQUITY UPLC H-Class liquid chromatograph.

The total polyphenols were extracted in the freeze-dried feed samples and quantified following the Folin–Ciocalteu reaction, according to Rufino-Moya et al. [30]. Samples and standard calibration were measured with a Heλios β spectrophotometer (Thermo Electron Corporation, Waltham, MA, USA) at 725 nm, and polyphenol contents were expressed as tannic acid equivalents.

### 2.5. Lipid Oxidation Analysis 

In every meat sample, lipid oxidation was determined by measuring the concentration of malondialdehyde (MDA) by liquid chromatography, using the procedure described by Bertolín et al. [31]. Briefly, 10 g of the meat samples were homogenized with 10 mL of 10% (*w*/*v*) aqueous trichloroacetic acid in ultrapure water and 50 μL of 7.2% (*w*/*v*) BHT in ethanol for 45 s with a high-performance homogenizer (Miccra D-8 Homogenizer, Falc Instruments, Treviglio, Italy) into a 50 mL polypropylene tube. The homogenizer was cleaned with 10 mL of 10% (*w*/*v*) aqueous trichloroacetic acid in ultrapure water, collecting the solution in the tube. Subsequently, the mixture was centrifuged for 15 min at 4000 rpm and 4 °C. Then, it was filtered through a paper filter, collecting the extract. A total of 1 mL of the vortexed extract was mixed with 10 mM 2-thiobarbituric (TBA). The mixture was homogenized and heated through agitation (45 min, 100 rpm at 100 °C) to form MDA-TBA_2_. After cooling, 150 μL was pipetted into a 2 mL amber screw-cap vial with 850 μL of a mixture of ACN:ultrapure water at a ratio of 30:70 (*v*:*v*). Finally, the extracts were injected into an ACQUITY UPLC H-Class liquid chromatograph (Waters, Milford, MA, USA).

### 2.6. Statistical Analysis 

The statistical analyses were conducted using Infostat software (version 2020, [32]). Drip loss, colour parameters, and MDA data were analysed with mixed models with repeated measures, where diet, display time, interaction diet x display time, and batch were considered as the fixed effects and individual as the random effect. The model was as follows:

$$y_{ijk}=\mu +\alpha_{i}+T_{j}+\beta_{m}+{\left(\alpha \times T\right)}_{ij}+L_{k}\left(a_{i}\right)+\varepsilon_{ijmkl}$$

where $y_{ijk}=$ dependent variable, μ= overall mean, $\alpha_{i}=$ fixed effect of carob inclusion level (i = C0%, C15%, C30%), $T_{j}=$ fixed effect of display time (j = 0, 7, 9, 11),$\beta_{m}=$ fixed effect of batch (m = winter, summer), ${\left(\alpha \times T\right)}_{ij}$= the interaction between diet and display time, $L_{k}\left(a_{i}\right)=$ the random effect of the individual nested within the diet, and $\varepsilon_{ijmkl}=$ residual error.

Likewise, the FA content and profile and tocopherol isomer and cholesterol contents in the raw meat were analysed through the standard least squares model: $y_{ijk}=\mu +\alpha_{i}+\beta_{j}+\varepsilon_{ij}$, where $y_{ijk}=$ dependent variable, μ= overall mean, $\alpha_{i}=$ carob inclusion level effect (i = C0%, C15%, C30%), $\beta_{j}=$ batch effect (j = winter, summer), and $\varepsilon_{ij}=$ residual error. The results are reported as the least squares means and their associated standard error of the mean (SEM). Comparisons between treatments were performed by Tukey’s test when significant (*p* < 0.05) effects were detected. Additionally, a *p*-value between 0.05 and 0.10 was considered a tendency. In all cases, the interaction between diet and batch was tested, and no significant effect was found; thus, it was excluded from the final model. 

Additionally, the α-tocopherol content of SM was segmented into seven categories with a range of 0.38 mg/kg meat from 1.16 to 3.82 mg/kg of meat (1.16–1.54; 1.54–1.92; 1.92–2.30; 2.30–2.68; 2.68–3.06; 3.06–3.44; and 3.44–3.82) to study the effect of meat α-tocopherol content on MDA formation at different display times. A mixed model with repeated measures was used, including display time and α-tocopherol categories as the fixed effects and individual as the random effect.

## 3. Results and Discussion

The batch effect was considered in the model, but the *p*-value and the least squares means for batch effect (winter vs. summer) are presented separately in this study (Supplementary Materials, Tables S1–S4), whereas the effects of dietary treatment, display time, and interaction between diet and display time are shown below.

### 3.1. Thawed and Drip Losses and Meat Colour Evolution

Interactions between diet and display time were found neither on colour parameters (L*, a*, b*, C*, h°) nor MMb formation nor on technological meat quality parameters, such as thawing loss (*p* > 0.05) or drip loss (*p >* 0.05). The moisture content was not affected by any factor; thus, the mean value was 76.1 ± 0.16%, regardless of treatment. Neither display time (*p* > 0.05) nor diet (*p* > 0.05) effects were found on drip loss (1.6 ± 0.2%; Supplementary Materials, Table S5). 

The impact of display time and later freezing on lamb meat quality have not been extensively reported in the literature. However, regardless of the diet, the effect of storage time (*p* < 0.01) was observed on the subsequent thawing losses, which were higher in meat stored for 0 and 7 days than 11 days (5.10 vs. 4.08 ± 0.29%), and intermediate in those stored for 9 days (4.68 ± 0.29%). Coincidently, Kim et al. [33] observed, in the beef loin, that the longer the aging period, the lower the thawing losses, which is possibly due to the low quantity of free water available after storage. 

**Figure 2:**
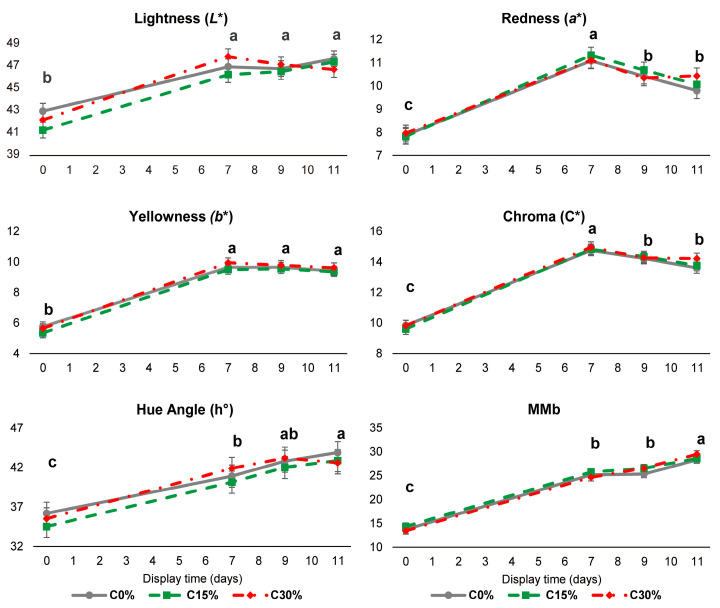
Evolution of the instrumental colour (lightness (L*), redness (a*), yellowness (b*), chroma (C*), hue angle (h°), and metmyoglobin (MMb)) of the meat of lambs fed with different carob pulp levels in their diet (0, 15, and 30%). Values are expressed as the mean ± standard error bars. Within a parameter, different letters denote significant differences between display days (*p* < 0.05).

Meat colour is one of the main aspects considered by the consumer in the purchase decision since a bright red colour is intuitively associated with the freshness and quality of the meat [34]. Display time, but not diet, affected meat colour attributes (Figure 2). In line with this, Gravador et al. [35] did not find any interaction between Cp and storage time, although they observed that diets with 35% of Cp produced darker (lower L*), less yellow (b*), and less saturated colour (C*) meat versus a control diet in heavy lambs (157-day old). 

In the current study, regardless of the diet, the L* and b* parameters showed the lowest level (*p* < 0.001) on day 0 and reached the highest value after day 7. Concomitantly, the redness (a*) and C* increased (*p* < 0.001) from the minimum value on day 0 to the highest level on day 7 and decreased to intermediate values on days 9 and 11. It was also observed that MMb and h° increased with storage time under MAP, achieving their maximum on day 11 (*p* < 0.001) and intermediate values on days 7 and 9. These results mirror the meat discolouration process from day 9, which is produced by the oxidation of myoglobin across the storage time and is generally reflected in a decrease in redness and saturation, and an increase in metmyoglobin levels and hue angle [36,37]. The browning of meat is the outcome of the oxidation of myoglobin to metmyoglobin, so when meat reaches 50% MMb, it appears reddish-brown and loses its sale value [3]. However, in this study, the MMb values ranged between 20 and 35%, which represents a small discolouration [18].

### 3.2. Fatty Acid Profile in Semimembranosus Muscle

The lamb meat FA composition and content may be affected by dietary FA profile, animal breed, sex, and age [37], or even by the amount of dietary polyphenols supplied, especially when lambs are fed with tannin-rich ingredients [9]. In this study, all diets were highly concentrated in nutrients and formulated to meet the recommended net energy level for Spanish fattening lambs [38]. Thus, it is worth noting that diets including Cp presented a higher percentage of ether extract since different amounts of oils were needed to formulate isonitrogenous and isoenergetic diets because fibrous ingredients, such as Cp, were supplied in the diets. 

Feeds with Cp tended to increase the total FA content in lambs (*p* = 0.058, Table 2). Gravador et al. [35] also provided Cp (35%) in diets to lambs but did not find differences on IMF in their loin compared with the control lambs, probably because the concentrates used presented similar ether extract. It is known that the IMF content is a key determinant of quality in meat because, within moderate levels, there is a positive impact on flavour and juiciness [39]. However, some recent meta-analyses found no effect of dietary inclusion of CT-rich feedstuff on the IMF of lamb meat [9,40]. Contrary, other studies reported a negative effect of carob on fat content in the carcass or meat from lambs [5]. In our experiment, and in addition to the IMF content results obtained, carcass dressing was unaffected by including up to 30% of Cp in concentrate [17]. Thus, the carob-fed lambs met their dietary energy requirements and, thereby, the potential antinutritional factors of Cp were counterbalanced.

The inclusion of Cp in the lamb concentrate affected the FA composition of IMF; the amounts of FA expressed by 100 g of meat are shown in Table 2 and Table 3. Although many studies on the FA content of meat present it as the percentage of each FA to the total FAME (i.e., % of total FA), to our understanding, the current expression is appropriate to assess the concentration of the rapidly and truly oxidizable FA per unit of muscle and consequently its oxidative sensitivity (more detailed information on the FA profile expressed as g FA/100 g FAME is available in the Supplementary Materials, Table S6). Meat from Cp-fed lambs tended to show a higher amount of C16:0 (*p* = 0.052), C22:0 (*p* = 0.051), and total saturated FAs (SFA, *p* = 0.077) compared with C0% lambs. In addition, the C18:0 amount was higher in C30% than in C0% lambs (*p* < 0.05). It is worth noting that lambs from the C15% and C30% groups ingested a higher level of SFA, specifically C16:0, compared to C0% individuals, which might explain the greater muscle deposition of those FA in the Cp lambs. Within the SFA series, there is an increasing interest in analysing the odd- and branched-chain FA (OBCFA). These FA are considered as potential indicators of the abundance and activity of microbial groups, and therefore may reflect the effects of the chemical composition of the concentrate in the ruminal environment. The negative effects of secondary compounds (i.e., CTs) have been reported on the OBCFA concentrations in digesta or even on volatile FA concentration, suggesting modifications in bacterial activity [6,41]. These previous findings support our observations, since lambs from the Cp groups tended (*p* = 0.059) to have decreased levels of C15:0 in IMF. Additionally, lambs from the C30% group exhibited a lower sum of iso-OBCFA and total OBCFA compared to both C15% and C0% lambs (*p* < 0.05). These results clearly suggest that the dietary inclusion of Cp impacted on ruminal microbiota activity, subsequently influencing the deposition of microbial-origin FA in the meat. In this study, the lower the inclusion of barley in the C30% treatment, the lower the OBCFA deposited in the IMF of lambs fed with 30% Cp. The previous literature has shown that a higher concentration of ruminal propionate leads to a greater concentration of OBCFA in the subcutaneous fat of sheep [42]. Thus, the lower starch content in the C30% concentrate of our study likely affected the FA synthesised from amylolytic- or starch- and sugar-digesting bacteria [43,44]. Natalello et al. [45] also confirmed a decrease in the OBCFA content of the digesta, liver, and meat in lambs fed with whole pomegranate, which is a rich-tannin Mediterranean by-product.

Additionally, the production of ruminal propionate decreases when vegetable oils are supplemented in diets [46]. Considering that the C30% diet had the highest level of vegetable oil inclusion, this factor may also explain the decrease in OBCFA levels in the IMF.

The sum of monounsaturated fatty acids (MUFA, Table 3) and the *cis*-MUFA content tended to be higher in C30% (*p* = 0.088 and 0.086, respectively) compared to C0%, as the oleic acid (*cis*9-C18:1) is the major MUFA in lamb meat, as it was already documented [8]. The PUFA content of the meat increased (*p* < 0.05) linearly in the lambs treated with Cp. Thereby, meat from the C30% group showed a higher amount of PUFA than the C0% group. Diaz et al. [37] suggested that SFA and MUFA levels increase faster than those of PUFA as fat deposition increases, leading to a reduction in the relative proportion of PUFA. Nevertheless, in the present study, the relative proportion of the total PUFA (15.85 ± 0.66 g PUFA/100 g FAMEs, Table S6) was similar among treatments (*p* > 0.05), although the total FAs tended to increase in C30%. These results may be explained by two causes. Firstly, a higher ingestion of fat in the Cp diets led directly to an increase in the PUFA meat content, such as linoleic acid (C18:2n-6), which was also higher (*p* < 0.05) in the C30% compared to the C0% group. A second explanation might be related to the presence of secondary metabolites in Cp, which could inhibit ruminal BH [10,47]. Some authors support the idea that a low content of VA (*trans*11-C18:1) in ruminant meat is a good indicator of a disrupted BH process, as a result of the CTs supplied [47]. However, it depends on which pathway of the BH is affected, because a higher VA content may be expected when the last step of BH (VA to SFA) is impaired [14]. In any case, in this paper, no differences were found among treatments (*p* > 0.05) on *trans*11-C18:1 or in rumenic acid (*cis*9, *trans*11-C18:2), which is another BH intermediary. These results seem to indicate that the level of CTs supplied did not extensively modify the BH. On the other hand, there is an alteration in the BH process known as “*t*-10 shift”, which occurs in concentrate-fed ruminants and consists of an accumulation of *trans*10-C18:1 instead of *trans*11-C18:1 in the rumen [48]. *trans*10-C18:1 is related to cardiovascular diseases in humans; thus, the 10 *trans*/11 *trans* ratio close to or lower than one is recommended as an indicator of the good quality of ruminant products [24]. In this study, the 10 *t*/11 *t* ratio was lower (*p* < 0.05) in the C30% compared to the C15% group and intermediate in the C0% group. Natalello et al. [45] observed a similar situation in lambs fed with a pomegranate by-product and attributed the results to the greater ingestion of fibre in those diets. The C30% diets were more fibrous (i.e., higher acid-detergent fibre and lignin content) and contained less starch, which is due to the Cp chemical composition [17].

### 3.3. Tocopherol and Cholesterol Contents in the Semimembranosus Muscle

The meat content of γ- and δ-tocopherol was similar among treatments (*p* > 0.05, 154 ± 13.4 and 6.32 ± 0.40 ng/g meat, respectively), although in the experimental concentrate, the higher the Cp content, the higher the γ-tocopherols level (Table 1). α-tocopherol is the most biologically active antioxidant stereoisomer of vitamin E, and in this paper, its dietary concentration was negatively affected by Cp inclusion. Despite the presence of a similar level of the in-feed supplemented vitamin E (300 IU/kg of feed), variations in the proportion of ingredients influenced the total α-tocopherol content in the concentrates, since barley (20 mg of α-tocopherol/kg of DM [49]) was the main ingredient replaced by Cp (4.05 mg of α-tocopherol/kg of DM). Consequently, the α-tocopherol content in the meat from the lambs without dietary Cp was higher (*p* < 0.05) than that in the C30% group (2.83 vs. 2.30 ± 0.16 mg/kg) and intermediate in C15% (2.41 ± 0.16 mg/kg). These results are supported by the fact that the magnitude of muscle vitamin E depends on the amount and chemical form and the length of the vitamin supplementation [50,51]. Thus, it is important also to point out whether the lambs studied were light (<25 kg of BW) or heavy, which influences the IMF level, or whether they were fed on pastures or indoors with concentrates and straw [52]. Contrary to our results, Lobón et al. [53] found that the dietary inclusion of condensed tannins in sheep increased the concentration of vitamin E in the meat from their suckling lambs. In addition, Valenti et al. [54] found an increase in meat α-tocopherol content in lambs fed with a tannin extract. However, in this latter study, the fact that no vitamin E was supplemented and the supplied tannins were mainly hydrolysable might be responsible for their findings. 

Our results do not confirm the effects mentioned of these secondary compounds on the vitamin E deposition. Nevertheless, it is also possible to hypothesize that the higher level of IMF (*p* = 0.058, tendency) observed in the C30% exerted a diluting effect on the meat α-tocopherol content.

Cp inclusion in the lamb’s concentrate had no impact on the cholesterol content in the meat (*p* > 0.05, 84.5 ± 0.3 mg/100 g meat), which coincides with previous works on heavy lambs supplemented with tannin extracts [55].

### 3.4. Lipid Oxidation

**Figure 3:**
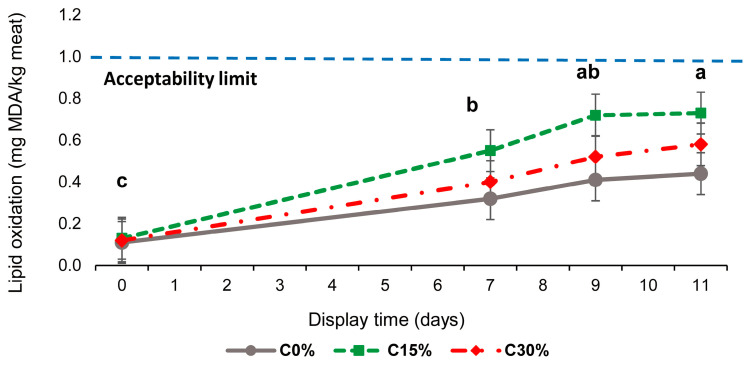
Effects of dietary carob pulp levels (0, 15, and 30%) on the lipid oxidation of lamb meat. The error bars represent standard error. Letters (a, b, and c) indicate significant differences between the display times (*p* < 0.05).

Lipid oxidation was affected by neither the diet and display time interaction nor dietary treatments (*p* > 0.05), which is in accordance with previous studies including dietary tannins [7,35,54]. Nevertheless, it is noteworthy that there is literature supporting the use of feedstuff rich in phenolic compounds to improve oxidative stability in meat [56]. MDA, which is considered a good indicator of the development of rancid off-flavours, increased linearly until 9 days of display, regardless of diet (*p* < 0.001) (Figure 3).

In light lambs, Ripoll et al. [57] showed that a level above 1 mg MDA/kg of meat seems to be the threshold to detect the oxidised flavour for meat. In this paper, the MDA mean concentrations remained far below this limit, even at 11 days of display. Previous studies showed that lipid oxidation products (i.e., aldehydes) decrease meat colour stability by influencing the enzymes involved in metmyoglobin reduction [7]. Recently, a meta-analysis [39] concluded that dietary CTs may preserve the antioxidant capacity and reduce lipid oxidation only if the deposition of pro-oxidant agents (such as PUFA) does not break the balance provided by antioxidant agents. Furthermore, it has been proposed that dietary phenolic compounds might positively interact with antioxidants, such as vitamin E, protecting and increasing their muscle concentration [51] or even being restored from their oxidized form [14]. Thus, the lack of dietary effect on MDA levels, despite the lower initial α-tocopherol muscle content in C30% lambs, might be associated with the potential capacity of CTs to regenerate α-tocopherols via the one-electron reduction of α-tocopheroxyl radicals [58]. However, the current results may not completely confirm this idea; thus, it is feasible to hypothesize that the level of total polyphenols supplied in the experimental Cp diets (8–12.1 g tannic acid equivalents/kg of Cp concentrate) could limit meat deterioration, which may counterbalance the above-mentioned pro-oxidant conditions in meat.

### 3.5. Relationship between α-Tocopherol Meat Content and Lipid Oxidation

**Figure 4:**
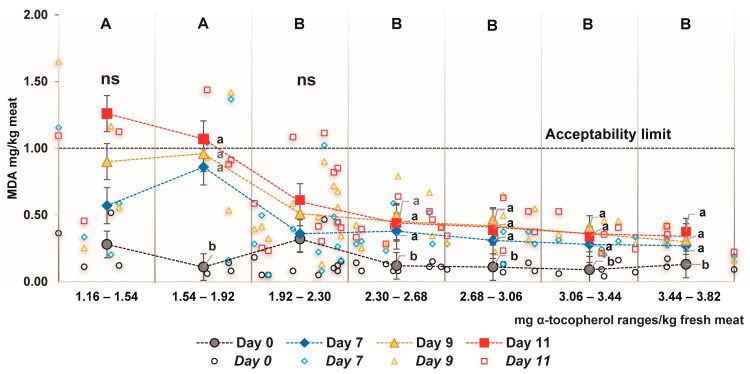
Relationship between the α-tocopherol content and lipid oxidation (MDA) in the *Semimembranosu*s muscle. Mean (±standard error) bars are indicated with solid symbols, while empty symbols indicate observational data for each display day. Different letters indicate significant differences (*p* < 0.05) between the overall α-tocopherol ranges at day 0 (capital letters) and days of display within each range (lowercase letters). ns: *p* > 0.05.

The meat content of α-tocopherol in all the samples ranged between 1.16 and 3.82 mg/kg of meat, but it is still unclear which is the optimal concentration in the tissue to limit the oxidative process. Previous studies in light lamb meat stored for 7 days [59] and 14 days [60] found that concentrations around 0.75 and 1.90 mg of α-tocopherol/kg of meat, respectively, were optimal thresholds above which the ability to stop the browning process and lipid oxidation was less efficient. In this study, seven intervals of α-tocopherol (0.38 mg/kg meat in each) were established with the aim of identifying the above-mentioned threshold. It is important to note that, for this analysis, the α-tocopherol content detected in the meat on day 0 was used; so, the continuing rate of vitamin E loss in the muscle was not considered. Two intervals (1.16–1.54 and 1.54–1.92 mg of α-tocopherol/kg meat) were statistically different (*p* < 0.05) and showed the highest MDA value compared with the other five ranges (Figure 4). Therefore, a concentration of 1.9–2.3 mg α-tocopherol/kg of meat was optimal, above which there was no detectable improvement in lipid oxidation disruption, even when the meat was displayed for 11 days under MAP conditions. In heavier lambs, 1.90 mg of α-tocopherol/kg meat also represented a turning point below which lipid oxidation started to increase rapidly in the meat stored in MAP [49]. 

## 4. Conclusions

The inclusion of Cp in the diets of light lambs up to 30% does not negatively affect meat quality parameters, such as drip loss or colour. However, our findings may not fully prove the idea that Cp limits the oxidative process in the *Semimembranosus* muscle, since meat discoloration and lipid oxidation were similarly increased in the meat from lambs consuming Cp compared to those that did not. The inclusion of 30% Cp in the lamb concentrate does not affect the SFA content, but results in a higher PUFA content, especially in the C18:3n-3 and n-6 series compared with the control diet. Unexpectedly, the α-tocopherol content in the meat was the lowest in the lambs fed with 30% Cp. Therefore, further research is needed to elucidate the relationship between dietary Cp inclusion and α-tocopherol metabolism and muscle deposition. Meanwhile, the concentration of vitamin E in the meat should not be lower than 2 mg α-tocopherol/kg to avoid lipid oxidation for up to 11 days of the display of meat from light lambs.

## Figures and Tables

**Table 1:** Means ± standard deviation of fatty acid composition and antioxidant and polyphenol contents in the feed (carob pulp; diets of 0, 150, and 300 g/kg of Cp; and barley straw).

	Carob Pulp	C0%	C15%	C30%	Barley Straw
Ether extract (% on DM basis)	1.81 ± 0.05	2.78 ± 0.27	5.00 ± 0.28	7.20 ± 0.19	2.71 ± 0.08
FA composition (g/100 g of FAME)					
C12:0	0.13 ± 0.02	0.18 ± 0.01	0.33 ± 0.02	0.38 ± 0.03	0.86 ± 0.06
C14:0	0.96 ± 0.15	0.50 ± 0.01	0.67 ± 0.00	0.81 ± 0.01	2.82 ± 0.01
C16:0	35.4 ± 0.25	25.6 ± 0.50	29.9 ± 0.58	34.9 ± 0.87	43.0 ± 0.52
C17:0	0.26 ± 0.02	0.11 ± 0.00	0.11 ± 0.00	0.11 ± 0.00	0.31 ± 0.04
C18:0	23.6 ± 2.91	6.40 ± 0.37	5.31 ± 0.38	5.65 ± 0.03	23.6 ± 2.16
C20:0	0.76 ± 0.01	0.35 ± 0.02	0.32 ± 0.02	0.35 ± 0.01	1.32 ± 0.05
C22:0	0.28 ± 0.05	0.14 ± 0.00	0.12 ± 0.02	0.10 ± 0.01	1.07 ± 0.13
C24:0	0.17 ± 0.01	0.12 ± 0.01	0.09 ± 0.02	0.08 ± 0.01	0.59 ± 0.13
Σ SFA	61.6 ± 2.50	33.4 ± 0.90	36.8 ± 1.00	42.4 ± 0.86	73.6 ± 1.98
*cis*9-C16:1	0.29 ± 0.41	0.14 ± 0.00	0.15 ± 0.01	0.16 ± 0.00	0.22 ± 0.00
*cis*9-C18:1	26.6 ± 1.80	2 3.5 ± 0.15	28.9 ± 0.06	33.1 ± 0.18	11.3 ± 1.35
*cis*11-C18:1	0.17 ± 0.10	0.79 ± 0.01	0.80 ± 0.01	0.80 ± 0.02	0.56 ± 0.05
C20:1	0.09 ± 0.05	0.23 ± 0.04	0.17 ± 0.00	0.14 ± 0.00	0.28 ± 0.40
Σ MUFA	27.1 ± 2.26	24.7 ± 0.11	30.0 ± 0.04	34.2 ± 0.16	12.4 ± 0.90
C18:2n-6	10.1 ± 0.23	39.2 ± 0.85	31.2 ± 0.99	22.1 ± 0.94	11.5 ± 1.42
C18:3n-3	1.17 ± 0.01	2.65 ± 0.14	1.96 ± 0.05	1.36 ± 0.08	2.52 ± 0.35
Σ PUFA	11.28 ± 0.24	41.9 ± 1.00	33.2 ± 1.03	23.4 ± 1.03	14.0 ± 1.07
n-6/n-3	8.69 ± 0.14	14.8 ± 0.48	15.9 ± 0.13	16.2 ± 0.27	4.66 ± 1.21
Antioxidant concentration (mg/kg DM)					
α-tocopherol	4.05 ± 0.62	251 ± 18.9	212 ± 14.1	213 ± 12.9	2.09 ± 0.85
γ-tocopherol	0.90 ± 0.17	7.44 ± 5.75	9.26 ± 5.34	11.7 ± 2.04	4.94 ± 6.01
δ-tocopherol	0.29 ± 0.02	1.58 ± 0.53	1.95 ± 0.34	1.80 ± 0.11	0.68 ± 0.77
Total polyphenols (g tannic acid eq/kg DM)	21.5 ± 0.28	8.00 ± 0.85	9.90 ± 0.86	12.1 ± 1.10	13.5 ± 1.40

DM: dry matter. FA: fatty acids. Σsaturated FA (SFA): C12:0 + C14:0 + C16:0 + C17:0 + C18:0 + C20:0 + C22:0+ C24:0. Σmonosaturated FA (MUFA): *cis*9-C16:1 + *cis*9-C18:1 + *cis*11-C18:1 + C20:1. Σpolyunsaturated FA (PUFA): C18:2n-6 + C18:3n-3.

**Table 2:** Effect of dietary carob pulp levels (0, 15, and 30%) on the total FA content (mg FAME/100 g meat) and the composition of saturated fatty acids (mg FA/100 g meat) of the *Semimembranosus* muscle.

	C0%	C15%	C30%	SEM ^1^	*p*-Value
*Total FA*	1800	2138	2293	0.15	0.058
C10:0	1.05	1.52	1.23	0.34	0.628
C11:0	0.16	0.2	0.19	0.04	0.665
C12:0	4.31	4.83	4.63	0.53	0.784
C13:0	11.1	10.5	9.50	0.56	0.122
C14:0	58.5	66.9	63	6.25	0.642
C15:0	17.3	17.2	14.9	0.78	0.059
C16:0	432	522	560	36.2	0.052
C17:0	32.7	34.1	33.0	2.60	0.916
C18:0	252 ^b^	303 ^ab^	330 ^a^	19.7	0.029
C19:0	0.66	1.02	0.97	0.14	0.165
C20:0	1.82	1.78	1.64	0.11	0.479
C21:0	0.65	0.96	0.85	0.11	0.185
C22:0	0.45	0.63	0.86	0.11	0.051
C24:0	0.07	0.08	0.08	0.02	0.950
∑*iso-*OBCFA	27.9 ^a^	28.1 ^a^	24.5 ^b^	1.10	0.041
∑*anteiso-*OBCFA	13.09	13.12	11.06	0.76	0.096
∑OBCFA	41.0 ^a^	41.2 ^a^	35.5 ^b^	1.78	0.047
∑DMA	60.3 ^b^	72.6 ^ab^	91.5 ^a^	7.26	0.016
∑SFA Me	11.5	12.8	13.3	0.91	0.418
∑SFA	813	967	1022	63.8	0.077

^1^ Standard error of means. ^a,b^ Least squares means in the same row with different superscript letters are different (*p* < 0.05). ∑OBCFA = sum of anteiso (*a*)- and iso (*i*)-fatty acids. ∑DMA = sum of dimethylacetals (DMA-C16:0 + DMA-C18:0 + DMA-C18:1). ∑SFA Me = ∑C14:0Me (C14:0-6Me + C14:0-8Me + C14:0-4Me + C14:0-10Me + C14:0-2.6DiMe) + ∑C15:0Me (C15:0-8Me + C15:0-4Me) + ∑C16:0Me (C16:0-2Me + C16:0-6Me + C16:0-8Me + C16:0-4Me + C16:0-12Me) + ∑C17:0Me (C17:0-12Me + C17:0-cyclo).

**Table 3:** Effects of dietary carob pulp levels (0, 15, and 30%) on the monounsaturated (MUFA) and polyunsaturated fatty acid (PUFA) compositions (mg FA/100 g meat) of the *Semimembranosus* muscle.

	C0%	C15%	C30%	SEM ^1^	*p*-Value
C12:1	0.28	0.23	0.27	0.03	0.411
C14:1	2.19	2.03	1.84	0.17	0.389
C15:1	1.89	2.11	2.15	0.17	0.512
C16:1	36.0	39.5	43.2	3.52	0.368
*cis*-C16:1	32.3	35.7	39.4	3.50	0.332
*trans*-C16:1	3.70	3.76	3.77	0.33	0.985
C17:1	27.1	28.9	34.5	2.48	0.101
*cis-*C17:1	25.6	27.4	33.3	2.59	0.083
*trans*-C17:1	1.47	1.52	1.26	0.12	0.264
C18:1	640	765	837	59.4	0.080
*cis*9-C18:1	528	619	685	52.0	0.098
*cis11-*C18:1	41.9 ^b^	51.7 ^ab^	63.6 ^a^	5.66	0.026
*trans*10*-*C18:1	24.8	34.4	27.2	3.73	0.150
*trans*11*-*C18:1	20.6	27.0	26.9	3.08	0.240
*trans*10/11*-*C18:1	1.26 ^ab^	1.33 ^b^	1.01 ^a^	0.09	0.041
C20:1	1.66	1.59	1.48	0.12	0.530
C22:1	0.54	0.46	0.55	0.05	0.272
C24:1	0.11	0.10	0.10	0.02	0.930
∑*cis-*MUFA	654	767	857	61.5	0.086
∑*trans-*MUFA	54.7	72.4	64.4	6.71	0.200
∑MUFA	709	840	921	65.0	0.088
C18:2	159 ^b^	191 ^ab^	200 ^a^	11.0	0.038
C18:2n-6	153 ^b^	184 ^ab^	192 ^a^	10.4	0.039
C19:2n-6	0.9	0.77	0.67	0.08	0.123
C18:3n-6	1.41	1.54	1.53	0.10	0.620
C18:3n-3	7.13 ^b^	8.41 ^ab^	9.42 ^a^	0.58	0.035
C20:2n-6	1.43	1.39	1.33	0.09	0.744
C20:3n-9	11.9	12.8	14.8	0.95	0.111
C20:3n-6	5.32	5.67	5.82	0.26	0.397
C20:3n-3	0.08	0.11	0.06	0.01	0.081
C20:4n-6 ARA	62.5 ^b^	72.5 ^ab^	77.7 ^a^	3.91	0.033
C20:5n-3 EPA	6.02	6.92	7.43	0.72	0.397
C22:4n-6	4.79	5.10	5.07	0.26	0.653
C22:5n-6	0.84 ^b^	1.27 ^ab^	1.37 ^a^	0.14	0.027
C22:5n-3 DPA	10.7	11.8	12.2	0.55	0.173
C22:6n-3 DHA	4.39	5.1	5.02	0.47	0.530
∑ CLA	7.48	7.48	7.88	0.50	0.806
*cis*9, *trans*11-C18:2	3.72	3.34	3.41	0.35	0.741
∑ n-6	230 ^b^	272 ^ab^	286 ^a^	14.3	0.030
∑ n-3	28.3	32.4	34.1	2.10	0.164
∑ PUFA	284 ^b^	332 ^ab^	350 ^a^	16.8	0.028
n-6/n-3	8.59	8.71	8.66	0.47	0.836
PUFA/SFA	0.36	0.35	0.35	0.37	0.842

^1^ Standard error of the mean. ^a,b^ Least squares means in the same row with different superscript letters are different (*p* < 0.05). ∑*cis-*MUFA = C12:1 + C14:1 + *cis*8-C15:1 + *cis*9-C15:1 + *cis*7-C16:1 + *cis*9-C16:1 + *cis*11-C16:1 + *cis*15-C16:1 + *cis*5-C17:1 + *cis*7-C17:1 + *cis*9-C17:1 + DMAC18:1 + *cis*11-C17:1 + *cis*6/*cis*8-C18:1 + *cis*9-C18:1 + *cis*11-C18:1 + *cis*12-C18:1 + *cis*13-C18:1 + *cis*14-C18:1 + *cis*15-C18:1 + C20:1 + C22:1 + C24:1. ∑*trans-*MUFA = *trans*5-C18:1 + *trans*6/*trans*8-C18:1 + *trans*9-C18:1 + *trans*10-C18:1 + *trans*11-C18:1 + *trans*12-C18:1. ∑CLA = Sum of conjugated linoleic acids.

## Data Availability

Data are contained within the article or Supplementary Materials.

## Supplementary Materials

The following supporting information can be downloaded at: https://www.mdpi.com/article/10.3390/antiox12081482/s1. Table S1: Effect of batch (winter or summer) on meat colour parameters (*L**; *a**; *b**; *C**, chroma; and h°, hue angle), metmyoglobin formation (% MMb), drip loss, and lipid oxidation (MDA, malondialdehyde mg/kg meat) on *Semimembranosus* muscle. Table S2: Effect of batch (winter or summer) on meat tocopherol (α, γ, and δ) and cholesterol content of *Semimembranosus* muscle. Table S3: Effect of batch (winter or summer) on total FAs (mg FAME/100 g meat) and the composition of saturated fatty acids (mg FA/100 g meat) of *Semimembranosus* muscle. Table S4: Effect of batch (winter and summer) on the composition of mono- and poly- unsaturated fatty acids (mg FA/100 g meat) of *Semimembranosus* muscle. Table S5: Effects of dietary carob pulp inclusion (0, 15, and 30%) in the lambs’ diet and display time (0, 7, 9, and 11 days) under Modified Atmosphere Packaging on the drip loss and thawed losses of lamb meat.Table S6: Effects of carob pulp levels (0, 15, and 30%) in the lambs’ diet on the fatty acid composition (g FA/100 g of FAMEs) of *Semimembranosus* muscle from light lambs.
