# Supplementary material for: Colour Stability, Fatty Acid Profile, and Lipid Oxidation in Meat Stored in Modified Atmosphere Packaging from Light Lambs Fed with Concentrate with Carob Pulp (Ceratonia siliqua L.)
Source: Antioxidants (Basel). 2023 Jul 25;12(8):1482. doi: 10.3390/antiox12081482 (PMC10451739; doi:10.3390/antiox12081482)
Supplement: Supplementary file 1 [file antioxidants-12-01482-s001.zip › antioxidants-2493460-supplementary.pdf]

antioxidants

MDPI

Supplementary material

Colour Stability, Fatty Acid Profile, and Lipid Oxidation in Meat Stored in Modified Atmosphere Packaging from Light Lambs Fed with Concentrate with Carob Pulp (*Ceratonia siliqua* L.)

Diego Nicolas Bottegal, Sandra Lobón; María Ángeles Latorre; Juan Ramón Bertolín; Javier Álvarez-Rodríguez.

Table S1. Effect of batch (winter or summer) on meat colour parameters (*L**; *a**; *b**; *C**, chroma; and h°, hue angle), metmyoglobin formation (%MMb), drip loss, and lipid oxidation (MDA, malondialdehyde mg/kg meat) on *Semimembranosus* muscle.

	Batch			
Item	Winter	Summer	SEM[1]	*p-value*[2]
*L**	44.8	46.8	0.40	<0.01
*a**	10.3	9.52	0.20	<0.01
*b**	8.40	8.77	0.18	0.159
*C**	13.4	13.0	0.21	0.141
h°	38.6	42.4	0.82	<0.01
%MMb	24.6	22.4	0.44	<0.01
Drip loss (%)	1.86	1.43	0.11	<0.01
Thawed loss (%)	3.50	5.96	0.29	<0.001
Moisture content (%)	76.0	76.0	0.16	0.886
MDA (mg/ kg meat)	0.41	0.42	0.05	0.945

[1] Standard error of mean.[2] Interaction between diet and batch was tested, and no significant effects were found.

Table S2. Effect of batch (winter or summer) on meat tocopherol (α, γ and δ) and cholesterol content of *Semimembranosus* muscle.

	Batch			
Item	Winter	Summer	SEM[1]	*p-value*
α-tocopherol μg/g meat	2.85	2.17	0.16	<0.001
γ -tocopherol ng/g meat	158	151	13.4	0.660
δ-tocopherol ng/g meat	6.95	6.67	0.40	0.564
Cholesterol mg/g meat	0.86	0.84	0.03	0.597

[1] Standard error of mean. [2] Interaction between diet and batch was tested, and no significant effects were found.

Supplementary material

Table S3. Effect of batch (winter or summer) on total FAs (mg FAME/100 g meat) and the composition of saturated fatty acids (mg FA/100 g meat) of *Semimembranosus* muscle.

Item	Batch		SEM[1]	*p*-value[2]
	Winter	Summer		
Total FA	2095	2063	113	0.842
C10:0	1.42	1.12	0.28	0.461
C11:0	0.17	0.19	0.03	0.665
C12:0	5.13	4.05	0.43	0.083
C13:0	10.5	10.3	0.45	0.792
C14:0	71.1	54.5	5.04	0.026
C15:0	17.1	15.8	0.63	0.156
C16:0	517	494	30.0	0.577
C17:0	32.8	33.7	2.12	0.761
C18:0	288	303	16.0	0.507
C19:0	0.86	0.91	0.12	0.758
C20:0	1.83	1.66	0.09	0.207
C21:0	0.84	0.81	0.10	0.811
C22:0	0.60	0.69	0.10	0.542
C24:0	0.10	0.06	0.02	0.138
∑iso-OBCFA	27.3	26.4	0.91	0.472
∑anteiso-OBCFA	13.1	11.7	0.62	0.116
∑OBCFA	40.4	38.1	1.50	0.262
∑DMA	71.2	73.6	6.00	0.979
∑SFA Me	12.4	12.1	0.75	0.672
∑SFA	947	921	52.0	0.718

[1]Standard error of mean. [2]Interaction between diet and batch was tested, and no significant effects were found. ΣOBCFA= sum of anteiso (*a*)- and iso (*i*)-fatty acids. ΣDMA=sum of dimethylacetals (DMA-C16:0 + DMA-C18:0 + DMA-C18:1). ΣSFA Me= ΣC14:0Me (C14:0-6Me + C14:0-8Me + C14:0-4Me + C14:0-10Me + C14:0-2.6DiMe) + ΣC15:0Me (C15:0-8Me + C15:0-4Me) + ΣC16:0Me (C16:0-2Me + C16:0-6Me + C16:0-8Me + C16:0-4Me + C16:0-12Me) + ΣC17:0Me (C17:0-12Me + C17:0-cyclo).

Table S4. Effect of batch (winter and summer) on the composition of mono- and poly- unsaturated fatty acids (mg FA/100 g meat) of *Semimembranosus* muscle.

Item	Batch		SEM[1]	*p*-value[2]
	Winter	Summer		
C12:1	0.29	0.23	0.03	0.142
C14:1	2.48	1.55	0.15	<0.001
C15:1	2.07	2.04	0.14	0.888
C16:1	43.3	35.7	2.90	0.069
cis-C16:1	39.7	31.8	2.70	0.047
trans-C16:1	3.62	3.87	0.26	0.501
C17:1	28.7	31.7	2.00	0.314
cis-C17:1	27.2	30.3	2.00	0.283

trans-C17:1	1.50	1.34	0.01	0.243
C18:1	742	752	49.0	0.871
*cis*9-C18:1	607	614	40.2	0.899
cis11-C18:1	50.8	54.0	4.38	0.615
*trans*10-C18:1	28.9	28.7	2.90	0.964
*trans*11-C18:1	24.4	25.3	2.40	0.795
*trans*10/11-C18:1	29.2	27.9	1.82	0.626
C20:1	1.53	1.62	0.09	0.495
C22:1	0.47	0.57	0.04	0.070
C24:1	0.11	0.10	0.02	0.772
∑*cis*-MUFA	756	762	50.2	0.938
∑*trans*-MUFA	63.8	63.9	5.5	0.990
∑MUFA	820	826	53.1	0.940
C18:2	184	182	9	0.837
C18:2n-6	177	175	8.69	0.836
C19:2n-6	0.81	0.75	0.06	0.475
C18:3n-6	1.45	1.53	0.08	0.495
C18:3n-3	9.13	7.51	0.48	0.022
C20:2n-6	1.42	1.35	0.07	0.467
C20:3n-9	12.6	13.8	0.78	0.320
C20:3n-6	5.75	5.46	0.2	0.322
C20:3n-3	0.07	0.09	0.01	0.166
C20:4n-6 ARA	71.9	70.0	3.2	0.681
C20:5n-3 EPA	7.94	5.64	0.58	0.009
C22:4n-6	4.51	5.46	0.21	<0.01
C22:5n-6	1.00	1.31	0.11	0.058
C22:5n-3 DPA	12.6	10.5	0.45	<0.01
C22:6n-3 DHA	5.80	3.87	0.39	<0.001
∑ CLA	7.91	7.32	0.41	0.314
*cis*9. *trans*11-C18:2	3.87	3.10	0.29	0.072
∑ n-6	264	261	11.68	0.833
∑ n-3	35.6	27.6	1.72	<0.01
∑ PUFA	327	316	13.7	0.570
n-6/n-3	7.59	9.72	0.39	<0.001
PUFA/SFA	0.36	0.35	0.01	0.819

[1]Standard error of mean. [2] Interaction between diet and batch was tested, and no significant effects were found. ∑*cis*-MUFA= C12:1 + C14:1 + *cis*8-C15:1 + *cis*9-C15:1 + *cis*7-C16:1 + *cis*9-C16:1 + *cis*11-C16:1 + *cis*15-C16:1 + *cis*5-C17:1 + *cis*7-C17:1 + *cis*9-C17:1 + DMAC18:1 + *cis*11-C17:1 + *cis*6/*cis*8-C18:1 + *cis*9-C18:1 + *cis*11-C18:1 + *cis*12-C18:1 + *cis*13-C18:1 + *cis*14-C18:1 + *cis*15-C18:1 + C20:1 + C22:1 + C24:1. ∑*trans*-MUFA=*trans*5-C18:1 + *trans*6/*trans*8-C18:1 + *trans*9-C18:1 + *trans*10-C18:1 + *trans*11-C18:1 + *trans*12-C18:1. ΣCLA= Sum of conjugated linoleic acids

Supplementary material

Table S5. Effects of dietary carob pulp inclusion (0, 15, and 30%) in the lambs' diet and display time (0, 7, 9, and 11 days) in Modified Atmosphere Packaging on the drip loss and thawed losses of lamb meat.

	Diet			SEM[1]	Display time (day)				SEM	*p*-value		
Item	C0%	C15%	C30%		0	7	9	11		Diet	Day	Diet*Day
Drip loss (%)	1.70	1.40	1.80	0.10	1.40	1.80	1.80	1.60	0.20	0.100	0.183	0.798
Thawed loss (%)	4.88	4.65	4.68	0.25	4.93[a]	5.26[a]	4.68[ab]	4.08[b]	0.29	0.363	0.004	0.084
Moisture content (%)	76.3	76.0	76.1	0.21	76.3	76.1	76.1	76.2	0.16	0.693	0.550	0.086

[1]Standard error of the mean. [a,b] Least square means in the same row with different superscript letters are different ($p < 0.05$)

antioxidants

MDPI

Table S6. Effects of carob pulp levels (0, 15, and 30%) in lambs' diet on the fatty acid composition (g FA/100 g of FAMEs) of *Semi-membranosus* muscle from light lambs.

Variable	C0%	C15%	C30%	SEM†	*p*-value
C10:0	0.06	0.08	0.05	0.02	0.606
C11:0	0.01	0.01	0.01	0.002	0.867
C12:0	0.24	0.22	0.2	0.02	0.472
C13:0	0.66[a]	0.54[ab]	0.44[b]	0.06	0.028
C14:0	3.18	3.1	2.74	0.19	0.183
C15:0	1.00[a]	0.85[ab]	0.67[b]	0.06	0.003
C16:0	23.8	24.4	24.3	0.27	0.199
C17:0	1.80[a]	1.58[b]	1.44[b]	0.05	<0.0001
C18:0	14.0	14.2	14.4	0.29	0.597
C19:0	0.04	0.04	0.04	0.01	0.577
C20:0	0.10[a]	0.09[ab]	0.07[b]	0.01	0.012
C21:0	0.04	0.05	0.04	0.01	0.494
C22:0	0.02	0.03	0.04	0.005	0.156
C24:0	0.004	0.005	0.004	0.001	0.869
∑BCFA	2.38[a]	2.04[ab]	1.6[b]	0.150	0.004
∑DMA	3.35	3.4	3.99	0.26	0.177
∑SFA Me	0.64[a]	0.62[ab]	0.56[b]	0.02	0.008
∑SFA	45.0	45.2	44.5	0.57	0.656
C12:1	0.02	0.01	0.01	0.002	0.150
C14:1	0.12[a]	0.10[ab]	0.08[b]	0.01	0.011
C15:1	0.11	0.1	0.1	0.01	0.639
C16:1	1.94	1.82	1.86	0.07	0.473
trans-C16:1	0.21	0.18	0.17	0.01	0.087
cis-C16:1	1.73	1.65	1.7	0.07	0.644

C17:1	1.49	1.34	1.51	0.07	0.153
trans-C17:1	0.08[a]	0.07[a]	0.06[b]	0.01	0.001
cis-C17:1	1.4	1.27	1.45	0.07	0.146
C18:1	35.1	35.3	36.3	0.84	0.503
*cis*9-C18:1	28.9	28.7	29.7	0.78	0.320
*trans*10-18:1	1.43	1.61	1.2	0.150	0.195
*trans*11-18:1	1.15	1.23	1.18	0.100	0.871
*cis*11-C18:1	2.27[b]	2.36[ab]	2.74[a]	0.140	0.044
C20:1	0.10[a]	0.08[ab]	0.07[b]	0.01	0.028
C22:1	0.03	0.02	0.02	0.003	0.129
C24:1	0.01	0.005	0.005	0.001	0.461
∑*trans*-C18:1	2.98	3.26	2.75	0.28	0.389
∑*cis*-C18:1	32.0	32.0	33.5	0.84	0.320
∑*cis* MUFA	35.8	35.5	37.2	0.83	0.320
∑*trans* MUFA	3.1	3.36	2.84	0.26	0.367
∑MUFA	38.9	38.8	40.0	0.88	0.530
C18:2	9.07	9.16	8.79	0.44	0.805
C19:2n-6	0.05[a]	0.04[ab]	0.03[b]	0.01	0.037
C18:3n-6	0.08[a]	0.07[ab]	0.07[b]	0.004	0.057
C18:3n-3	0.40	0.40	0.41	0.02	0.802
C20:2n-6	0.08[a]	0.07[ab]	0.06[b]	0.01	0.021
C20:3n-9	0.67	0.61	0.67	0.05	0.572
C20:3n-6	0.31	0.28	0.26	0.02	0.121
C20:3n-3	0.005	0.010	0.003	0.001	0.081
C20:4n-6 ARA	3.55	3.51	3.45	0.2	0.941
C20:5n-3 EPA	0.33	0.33	0.33	0.03	0.977
C22:4n-6	0.28	0.25	0.23	0.02	0.142
C22:5n-6	0.05	0.06	0.06	0.01	0.132

C22:5n-3 DPA	0.61	0.58	0.54	0.04	0.422
C22:6n-3 DHA	0.24	0.25	0.22	0.02	0.715
∑CLA	0.42[a]	0.36[b]	0.35[b]	0.02	0.014
*cis*9,*trans*11- C18:2	0.21[a]	0.16[ab]	0.15[b]	0.01	0.010
∑n-6	13.1	13.1	12.6	0.6	0.760
∑n-3	1.59	1.57	1.51	0.09	0.800
∑PUFA	16.2	16.0	15.5	0.71	0.756

[†]Standard error of the mean. [a.b] Least square means in the same row with different superscript letters are different ($p < 0.05$); ΣCLA= Sum of Conjugated Linoleic Acids. Σ BCFA= sum of a- and i-fatty acids. Σ DMA= sum of dimethylacetals (DMA-C16:0 + DMA-C18:0 + DMA-C18:1). Σ SFA Me= Σ C14:0Me (C14:0-6Me + C14:0-8Me + C14:0-4Me + C14:0-10Me + C14:0-2.6DiMe) + Σ C15:0Me (C15:0-8Me + C15:0-4Me) + Σ C16:0Me (C16:0-2Me + C16:0-6Me + C16:0-8Me + C16:0-4Me + C16:0-12Me) + Σ C17:0Me (C17:0-12Me + C17:0-cyclo). ∑ cis-MUFA= 12:1 + 14:1+ *cis*8-15:1 + *cis*9-15:1 + *cis*7-16:1 + *cis*9-16:1 + *cis*11-16:1 + *cis*15-16:1 + *cis*5-17:1 + *cis*7-17:1 + *cis*9-17:1 + DMA18:1 + *cis*11-17:1 + *cis*6/*cis*8-18:1 + *cis*9-18:1 + *cis*11-18:1 + *cis*12-18:1 + *cis*13-18:1 + *cis*14-18:1 + *cis*15-18:1 + 20:1 + 22:1 + 24:1. ∑ trans-MUFA= *trans*5-C18:1 + *trans*6/*trans*8-C18:1 + *trans*9-C18:1 + *trans*10-C18:1 + *trans*11-C18:1 + *trans*12-C18:1
